# ANZAED eating disorder treatment principles and general clinical practice and training standards

**DOI:** 10.1186/s40337-020-00341-0

**Published:** 2020-11-10

**Authors:** Gabriella Heruc, Kim Hurst, Anjanette Casey, Kate Fleming, Jeremy Freeman, Anthea Fursland, Susan Hart, Shane Jeffrey, Rachel Knight, Michelle Roberton, Marion Roberts, Beth Shelton, Garalynne Stiles, Fiona Sutherland, Chris Thornton, Andrew Wallis, Tracey Wade

**Affiliations:** 1 Australia & New Zealand Academy for Eating Disorders, Sydney, Australia; 2 School of Medicine, Western Sydney University, Campbelltown, Australia; 3 Eating Disorders Service, Northern Sydney Local Health District, St Leonards, Australia; 4 School of Psychology, Griffith University, Gold Coast, Australia; 5 Eating Disorders Service, Robina Private Hospital, Robina, Australia; 6 Centre for Psychotherapy, Hunter New England Local Health District, Newcastle, Australia; 7 The Swan Centre, Perth, Australia; 8 Nutrition and Dietetics, St Vincent’s Hospital, Darlinghurst, Australia; 9 The Boden Collaboration of Obesity, Nutrition, Exercise and Eating Disorders, University of Sydney, Camperdown, Australia; 10 River Oak Health, Brisbane, Australia; 11 Royal Brisbane and Women’s Hospital, Brisbane, Australia; 12 Occupational Therapy, School of Health and Social Development, Faculty of Health, Deakin University, Geelong, Australia; 13 The Victorian Centre of Excellence in Eating Disorders, Melbourne, Australia; 14 Department of General Practice and Primary Health Care, Faculty of Medical & Health Sciences, University of Auckland, Auckland, New Zealand; 15 National Eating Disorders Collaboration, Sydney, Australia; 16 School of Sport, Exercise and Nutrition, College of Health, Massey University, Palmerston North, New Zealand; 17 The Mindful Dietitian, Melbourne, Australia; 18 The Redleaf Practice, Sydney, Australia; 19 Eating Disorders Service, Sydney Children’s Hospital Network, Sydney, Australia; 20 Blackbird Initiative, Órama Institute, Flinders University, Bedford Park, Australia

**Keywords:** Clinical practice, Dietetics, Dietitian, Eating disorder, Mental health professional, Psychological, Standards, Training, Treatment

## Abstract

**Introduction:**

Eating disorders are complex to manage, and there is limited guidance around the depth and breadth of knowledge, skills and experience required by treatment providers. The Australia & New Zealand Academy for Eating Disorders (ANZAED) convened an expert group of eating disorder researchers and clinicians to define the clinical practice and training standards recommended for mental health professionals and dietitians providing treatment for individuals with an eating disorder. General principles and clinical practice standards were first developed, after which separate mental health professional and dietitian standards were drafted and collated by the appropriate members of the expert group. The subsequent review process included four stages of consultation and document revision: (1) expert reviewers; (2) a face-to-face consultation workshop attended by approximately 100 health professionals working within the sector; (3) an extensive open access online consultation process; and (4) consultation with key professional and consumer/carer stakeholder organisations.

**Recommendations:**

The resulting paper outlines and describes the following eight eating disorder treatment principles: (1) early intervention is essential; (2) co-ordination of services is fundamental to all service models; (3) services must be evidence-based; (4) involvement of significant others in service provision is highly desirable; (5) a personalised treatment approach is required for all patients; (6) education and/or psychoeducation is included in all interventions; (7) multidisciplinary care is required and (8) a skilled workforce is necessary. Seven general clinical practice standards are also discussed, including: (1) diagnosis and assessment; (2) the multidisciplinary care team; (3) a positive therapeutic alliance; (4) knowledge of evidence-based treatment; (5) knowledge of levels of care; (6) relapse prevention; and (7) professional responsibility.

**Conclusions:**

These principles and standards provide guidance to professional training programs and service providers on the development of knowledge required as a foundation on which to build competent practice in the eating disorder field. Implementing these standards aims to bring treatment closer to best practice, and consequently improve treatment outcomes, reduce financial cost to patients and services and improve patient quality of life.

## Definitions

Mental health professional includes many types of health professionals working in mental health care, including psychologists, social workers, mental health nurses, occupational therapists and counsellors.

## Plain English summary

Little research exists around the practitioner knowledge and skill required for the treatment of eating disorders. To ensure best practice, patient safety and optimal patient outcomes in eating disorder management, health professionals need to demonstrate adequate knowledge through specialised training and experience. The Australia & New Zealand Academy for Eating Disorders (ANZAED) convened an expert group of eating disorder researchers and clinicians to define the clinical practice and training standards recommended for mental health professionals and dietitians providing treatment for individuals with an eating disorder. There was a four-stage review and consultation process including: [1] expert reviewers [2]; a face-to-face consultation workshop attended by approximately 100 health professionals working within the sector [3]; an extensive open access online consultation process; and [4] consultation with key professional and consumer/carer stakeholder organisations. These clinical practice and training standards are intended to bolster the eating disorder knowledge of mental health professionals and dietitians who are treating patients with an eating disorder. This paper outlines the knowledge required of mental health and dietetic professionals, to successfully respond, treat and manage eating disorders and promote a coordinated and consistent approach to professional development and training with the aim of service improvement. Implementing these practice and training standards aims to bring treatment closer to best practice, and consequently improve treatment outcomes, reduce financial cost to patients and services and improve patient quality of life.

## Introduction

Eating disorders are severe, complex and often debilitating mental health conditions, characterised by a severe and persistent disturbance in eating behaviour. Affecting approximately 16% of adults [1], 8–15% of adolescents [2] and children as young as 5 years [3], they are often associated with serious psychological [2, 4], social [5] and physical complications [4, 6, 7], poor quality of life [8–10], as well as a significantly higher mortality than the average population and among the highest for a psychiatric illness [11]. Due to the psychiatric and medical complexity, a well-coordinated multidisciplinary treatment is often required, placing significant financial burden on individuals, families, services and societies [10, 12]. The earliest possible access to evidence-based treatment delivered by a competent workforce is essential to minimise financial cost and maximise patients’ physical wellbeing, mental health and quality of life. Professionals with limited experience and a poor understanding of eating disorder management may risk causing harm to patients or delay recovery [13]. Thus, to ensure best practice, patient safety and optimal patient outcomes, health professionals need to demonstrate a breadth of knowledge, through training and experience, in managing severe and complex eating disorders [14, 15].

To date, tertiary health education programs have provided limited training in eating disorders, and graduates enter the workforce with inadequate skills needed to work in this field. The National Agenda for Eating Disorders in Australia identified that 97% of clinicians had received no or insufficient training in eating disorders to enable them to provide treatment with confidence [16]. The National Eating Disorder Collaboration (NEDC) therefore developed a set of eating disorder core competencies as a foundation for strengthening the workforce [17]. The current paper builds on the NEDC competencies by outlining the essential principles of treatment provision and minimum clinical practice and training standards recommended for mental health professionals and dietitians providing treatment in the field of eating disorders. The standards detail the knowledge, practical skills and experience required to competently manage and treat patients with an eating disorder, as well as the expectations and content needed in training programs to support clinicians attaining these standards.

These principles and standards are a project of the Australia & New Zealand Academy for Eating Disorders (ANZAED), the peak body for eating disorder professionals involved in research, prevention, treatment and advocacy in Australia and New Zealand. ANZAED fosters professional development and networking in the eating disorder field and provides leadership within the eating disorder sector. This project represents the work of the co-authors, a core expert group of clinicians and researchers, and incorporates sector-wide public consultation and feedback. The current paper aims to provide guidance on the important treatment principles and minimum general clinical practice and training standards for mental health and dietetic professionals who provide treatment to individuals with an eating disorder.

## Methods of practice and training standards development

ANZAED established two expert working groups – the mental health professional and dietitian groups – with representatives from around Australia and New Zealand. The groups included both clinicians and academics each with greater than ten years’ experience within the field of eating disorders. The mental health group included nine professionals with backgrounds including psychology, social work and occupational therapy, and eight dietitians made up the other group.

Each working group initially developed separate draft standards based on published evidence and clinical experience. These were then combined to create overall general clinical practice standards, as well as profession-specific practice and training standards, which were reviewed and refined by each respective working group, ANZAED’s Executive Committee, and expert advisors. The next draft version was then presented for public face-to-face consultation at the ANZAED 2019 Conference in Adelaide, with feedback received and incorporated from eating disorder professionals (*n* = 100). Following this, online consultation from the broader public was sought, with feedback received from international expert advisors, professional bodies (various disciplines) and consumer and carer groups with comments reviewed and integrated by the working groups. The final version was also reviewed by the NEDC Steering Committee and again by ANZAED’s Executive Committee. Consensus on all papers was reached through discussion by the authors and there was no identified conflict of interest. The resulting eight principles and seven general clinical practice standards are detailed below. The mental health-specific [18] and dietetic-specific [19] practice and training standards are published separately.

## Recommendations

### General principles

**Fig. 1:**
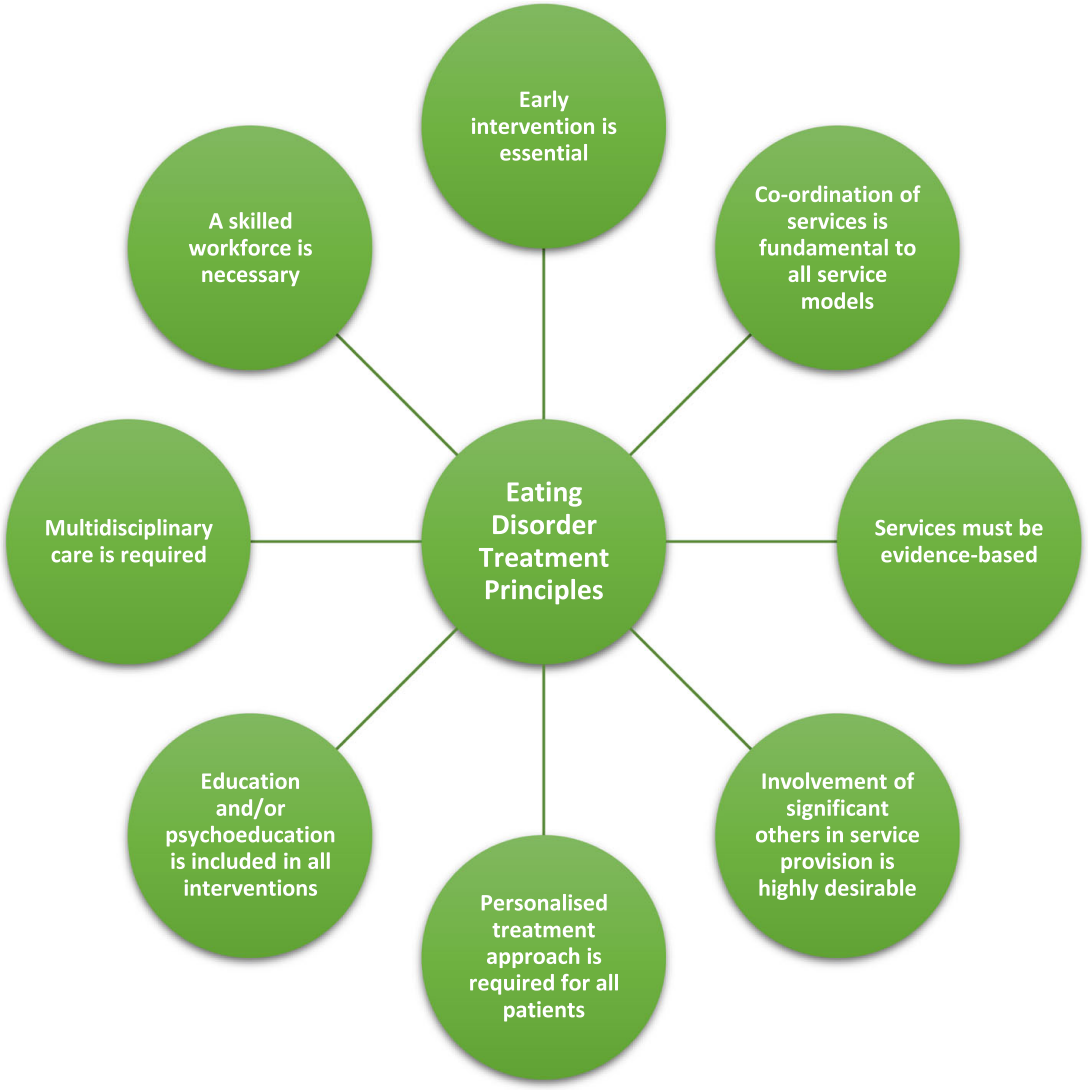
Eight eating disorder treatment principles for mental health professionals and dietitians

Patient journeys across the developmental spectrum of eating disorders are varying and complex, involving many different trajectories, from brief illnesses to recurring, but different eating disorders [20] each might present alongside various co-occurring diagnoses. Despite this complexity, the goal of recovery and improved quality of life informs all service provision. Recovery principles support individuals with an eating disorder, their family and significant others to engage in meaningful activities and occupations, undertake social activities, reinvest in important life roles, and restore and maintain appropriate eating habits, thoughts and behaviours. No single service model can be specified, but there are general principles that should guide the development and implementation of any service provision, as outlined in Fig. 1 and detailed below.

1. *Early intervention is essential.* Given the wide-ranging adverse impacts of an eating disorder on physical, mental, social and occupational health, aiming for the earliest intervention possible is necessary to prevent long-term suffering and disability [21]. Eligibility for services and treatments is not to be limited to the presence of an eating disorder that meets strict diagnostic criteria.

2. *Co-ordination of services is fundamental to all service models.* Disruption to care, and subsequent deterioration in the consumer’s health, may occur when individuals transition between treatment settings, e.g. inpatient to outpatient-based treatment, between public and private settings, or between child and adolescent services to adult services [22]. The co-ordination of eating disorder services that minimises or removes such disruptions is key to effective service provision.

3. *Services must be evidence-based.* The current recommendations for evidence-based therapy across different treatment guidelines is summarised in Table 1 [23]. Where deviations are considered necessary, options should be evaluated, and expert supervision sought. Deviations from evidence-based protocols are not uncommon [24], but most typically involve avoidance of effective elements of therapy that are perceived by clinicians as producing short-term distress for the patient [25, 26], and therefore cause the therapist anxiety. Inadequate treatment response to evidence-based eating disorder treatment may indicate a need for more intense treatment options [27], e.g. more frequent sessions at an earlier stage, use of adjunct therapies, the inclusion of family and significant others as part of the treatment team, day- or inpatient treatment, or multi-family therapy.
4. *Involvement of significant others in service provision is highly desirable.* Utilising the resources of the family and significant others, with consent, is a key pillar in many eating disorder treatments regardless of the age of the person and the type of eating disorder. In some treatments, family involvement is essential (e.g., Family therapy for child and adolescent anorexia nervosa). In addition, upskilling of significant others so they feel more confident and equipped to deal with an eating disorder while attending to their own needs is important [28].

5. *A personalised treatment approach is required for all patients.* The treatment intensity should be matched to the clinical presentation of the patient allowing for stepping up and down in intensity of care as needed, rather than automatically starting patients at the lowest intensity option. Clinician firmness and empathy are needed to promote change. Session by session evaluation [29] collaboratively shared with the patient (and family as appropriate) is essential, not only for improving outcomes [30], but also for detecting lack of early change as this predicts poorer outcome across eating disorders and modalities of treatment [31]. Services should be delivered in a culturally responsive manner, considering body size, age, sexuality and gender, with an awareness of working with Indigenous people (e.g. (Aboriginal & Torres Strait Islander, Maori [*tangata whenua*] and Pacific peoples) and people from culturally and linguistically diverse backgrounds. It is important that clinicians adopt a strengths-focused approach, supporting recovery and/or quality of life, tailored to meet individual decision-making capacity and needs as they develop over the course of the illness.
6. *Education and/or psychoeducation is included in all interventions.* Communication and information for those with an eating disorder, as well as families and significant others involved in supporting the person, is important, particularly given the degree of misinformation widely available about ‘healthy’ eating, weight and the medical impact of disordered eating. Education and/or psychoeducation should also include the rationale for evidence-based treatment strategies, including an initial focus on restoring nutritional health.

7. *Multidisciplinary care is required.* Eating disorders co-occur with a range of serious physical and psychological issues. Clinicians need to understand and ensure that a medical practitioner (e.g., general practitioner and/or paediatrician) is actively involved in patient care to provide regular medical monitoring and linked with other treating professionals, which may include psychiatrists, psychologists, dietitians, social workers, occupational therapists, mental health nurses and eating disorder-trained peer support workers. The clinician should be aware of and have access to tertiary-level eating disorder services, specialised private practices or professionals with relevant expertise for consultation, supervision, guidance and referral, if required.

8. *A skilled workforce is necessary.* Evidence suggests that clinician expertise in eating disorders produces better outcomes. For example, in child and adolescent anorexia nervosa, specialised outpatient eating disorder teams have been associated with faster recovery, higher patient satisfaction, lower costs, lower rates of inpatient admission and better case identification and access compared to care by generalist services [32, 33]. It is important, however, to note that expert supervision of novice therapists can produce similar outcomes to those of experienced therapists [34].

**Table 1:** Recommendations for treating eating disorders (ED) summarised from current treatment guidelines^a^ [23]

**General Principles** Treatment should include psychoeducation; weight monitoring; addressing physical and mental health (including self-harm or suicidal behaviour); involve a multidisciplinary team and coordinated care between services; include the person’s family members or significant others where appropriate; be mindful that individuals with an eating disorder are vulnerable to stigma and shame; be sensitive when discussing a person’s weight and appearance; assess and, where possible modify, the impact of the environment (e.g. social media).		
**Diagnosis**	**Principles specific to the eating disorder**	**Specific outpatient therapies recommended across the guidelines**
**Anorexia nervosa**	1. A key goal is to help patients reach a healthy body weight for their age 2. Explain to the patient and their family the effects of starvation on the brain and body, and the need to reverse starvation by nutritional rehabilitation 3. Weight gain is key to supporting other changes needed for recovery 4. When weighing consider sharing the results with the patient and family 5. Only offer dietary counselling as part of a multidisciplinary approach 6. Do not offer medication as a sole approach 7. Have clear criteria for moving to more intensive treatment, e.g. admission to hospital	**Children and adolescents:** Family therapy for anorexia nervosa ^b^ (parent-focused and multi-family group also acceptable); ED-focused CBT (CBT-ED) enhanced with family involvement; adolescent-focused psychotherapy **Adult:** Maudsley anorexia nervosa treatment for adults (MANTRA); Specialist supportive clinical management (SSCM); CBT-ED; eating disorder-focused focal psychodynamic therapy
**Bulimia nervosa**	1. Explain that psychological treatments have limited effect on body weight 2. Explain that dieting increases the chance of binge eating 3. Do not offer medication as a sole approach; SSRIs are recommended as an adjunct treatment	**Children and adolescents:** CBT-ED with family involvement; bulimia nervosa-focused family therapy **Adult:** Guided self-help CBT-ED; CBT-ED; interpersonal psychotherapy
**Binge eating disorder**	1. Explain that psychological treatments have limited effect on body weight 2. For adolescents, offer the same treatments as adults 3. Do not offer medication as a sole approach; SSRIs are recommended as an adjunct treatment	**Adolescents and adults:** Guided self-help CBT-ED; CBT-ED; interpersonal psychotherapy
**OSFED**: Offer the treatment for the eating disorder it most closely resembles; **ARFID:** Does not currently appear in treatment guidelines, the reader is referred to Thomas, J., & Eddy, K. (2019). *Cognitive-Behavioral Therapy for Avoidant/Restrictive Food Intake Disorder: Children, Adolescents, and Adults*. (2019). Cambridge: Cambridge University Press.		

^a^ Treatment guidelines included Royal Australian and New Zealand College of Psychiatrists Clinical Practice Guidelines for the Treatment of Eating Disorders, National Clinical Guidelines (Denmark), Clinical Practice Guidelines (France), S3-guidelines for Assessment and Treatment of Eating Disorders (Germany), Practice Guidelines for the Treatment of Eating Disorders (Netherlands), Clinical Practice Guidelines for Eating Disorders (Spain), Eating Disorders: Recognition and Treatment (United Kingdom), Practice Guidelines for the Treatment of Eating Disorders (USA)

^b^ Family therapy for AN also known as Family Based Treatment, Maudsley Family Therapy

### General clinical practice standards

To provide effective management of the physical and psychiatric complexities of eating disorders as well as clinical safety, minimum understanding, knowledge and skills are essential for health professionals practicing in the space. It is recommended that all clinicians involved in the assessment, diagnosis, management and treatment of patients with an eating disorder follow the below practice standards.

1. *Diagnosis and assessment.* Clinicians should be aware of and have a working knowledge of the current diagnostic criteria for eating disorders [35], and clinical features of related appearance and eating conditions. They should conduct an assessment consistent with their understanding and scope of practice and arrive at a shared understanding of the illness with their patient, family (if applicable) and treatment team.

2. *Multidisciplinary care team (MDT).* Treatment of eating disorders should be multidisciplinary, including a medical practitioner, mental health professional and a dietitian if accessible. Respective roles across the MDT should be clearly documented and understood, and a designated clinical lead identified. Processes of communication within the MDT need to be clearly outlined. All clinicians must practice within the scope of their profession and know when to refer to another clinician with focused eating disorder skills. However, all clinicians will need to have an interdisciplinary working knowledge of medical, mental health, nutritional and psychiatric aspects of eating disorders, as indicated below:

Medical: All clinicians should understand the significant physical risks associated with eating disorder behaviour, including the risk of death, and be aware of the parameters of physical stability, as outlined in the Royal Australian and New Zealand College of Psychiatry clinical practice guidelines for the treatment of eating disorders [36]. There is a need for all patients to have a medical assessment, preferably by a medical practitioner who understands the clinical symptoms and signs indicating the risks associated with eating disorders. Ongoing medical review should be a non-negotiable element of treatment.

Mental Health: All clinicians should understand core psychological principles including behaviour change, behavioural experiments, core counselling micro-skills, modifying cognitions, managing affect and addressing underlying issues that may maintain the behavioural aspects of the eating disorder. Referral to a mental health professional is recommended for most patients to provide psychological support and evidence-based psychological interventions.

Nutritional: All clinicians should have a knowledge of nutritional issues relevant to eating disorders (e.g. regular eating, the consequences of starvation or low energy availability, effects of binge eating and compensatory behaviours, body weight, paediatric growth charts [37], the importance of nutritional rehabilitation and an understanding of the importance of weight and health recovery). Referral to a dietitian experienced in eating disorder management is recommended for patients finding it difficult to achieve nutritional, behavioural or physical goals (e.g. with malnutrition, risk of refeeding syndrome, nutritional complications, etc.), or with co-occurring medical conditions that may affect nutritional management (e.g. pregnancy, diabetes, polycystic ovarian syndrome, food allergy/intolerance, bowel disease, etc.).

Psychiatric: All clinicians should have an awareness of common co-occurring psychiatric presentations, and the ability to assess and respond to a risk of harm to self, and suicidal ideation. An assessment by a psychiatrist, preferably one experienced in eating disorders, is recommended where risk is identified, a complex formulation and treatment plan is required and/or when medication is required to support complexity and co-occurring diagnoses.

3. *Positive therapeutic alliance.* Many people with an eating disorder find the process of change difficult. Clinicians should possess skills to develop a solid therapeutic alliance, address ambivalence about change and build up self-efficacy [38]. They should also reach a collaborative agreement on the approach to, and goals and topics of therapy. In addition the clinician should understand the need for non-negotiables and assist patients to achieve early symptom improvement, which enhances therapeutic alliance and treatment outcomes in eating disorders [39]. The clinician should be competent in managing the challenges that arise within the therapist-patient relationship, while utilising the support of family and significant others.

4. *Knowledge of evidence-based treatment.* Clinicians should be aware of clinical practice guidelines that summarise evidence-based approaches for the treatment of eating disorders (Table 1). At a minimum, treatment should be specific to the patient’s age, diagnosis and stage of illness. All clinicians should understand the roles and importance of general psychoeducation for eating disorders, regular in-session weighing (as appropriate), the establishment of regular eating, weight restoration and/or stability; self-monitoring and behavioural strategies directed at addressing behaviours such as restriction, binge eating, and compensatory behaviours. Where possible and appropriate, children or adolescents should be seen in a family-centred context, and adult patients’ families and significant others included in treatment.

5. *Knowledge of levels of care.* Clinicians should be aware of the local treatment options that may be available to their patient (e.g., acute medical hospitalisation, admission to a specialist eating disorder inpatient unit, a partial hospitalisation program or day program, intensive outpatient therapy or peer mentoring programs). It is important that clinicians are aware of the services that are not available in the local area, and to know where the closest available alternative treatment service is located. Treatment options may include involuntary treatment and the use of a community treatment order. Clinicians should be aware of local guidelines for moving between levels of care. Constant assessment of patient status and progress is needed to inform changes in the level of care. Wherever possible these changes should be made collaboratively with the patient. It should be recognised that psychotherapy with a severely malnourished patient (noting this can present across the weight spectrum) is unlikely to be effective in achieving behavioural change [36].

6. *Relapse prevention.* Every individual treated for an eating disorder requires a relapse management plan with monitoring for at least 12 months post-treatment [40]. An assertive relapse prevention program, developed with the patient, should remind them to come in for treatment where their own efforts are not sufficient to arrest deterioration.

7. *Professional responsibility.* Clinicians should be aware of and maintain important professional practices such as clinical supervision, professional development and practices to manage their own wellbeing. Clinical supervision and ongoing professional development aim to upskill clinicians, support reflective practice, aid the provision of high-quality treatment and recognise the intensity and personal impact of treating complex mental health issues. For mental health professionals and dietitians treating patients with an eating disorder, a significant component of these professional practices should be eating disorder-specific, incorporating the implementation of non-negotiables to establish boundaries around treatment. Clinicians should also be aware of their own attitudes toward body shape, weight, food and eating to avoid transmission of unhelpful messages or practices during therapy. It is expected that a clinician experiencing their own mental health difficulties while providing eating disorder treatment would seek appropriate support and modify their work as needed to maintain their own and their patients’ wellbeing.

### Implementation and future directions

Effective treatment of eating disorders typically requires a well-coordinated and skilled clinical management team across different disciplines [36] with a depth and breadth of knowledge and skills to achieve professional competence [41, 42]. The ANZAED treatment principles and clinical practice and training standards are the first of their kind for mental health professionals and dietitians in Australia and New Zealand. They outline the clinical skills, knowledge and experience required to competently manage patients with an eating disorder and can be used to guide and inform education and training programs.

Clinicians who assess and treat individuals with an eating disorder should be competent to do so [15]. Furthermore, they are responsible for monitoring their competence and treatment adherence, as well as ensuring current best practice. At present, there is no means in Australia or New Zealand to guarantee that professionals have adequate training and experience. However, these standards might form the basis of a credentialing system, providing guidance to professional training programs and service providers on the minimal standards required for appropriate and safe treatment delivery, as well as best patient outcomes. Applying the current principles and practice and training standards to individual treatment providers, treatment services and training providers will promote a more coordinated and consistent approach, bringing eating disorder treatment closer to best practice.

## Data Availability

Not applicable.
